# Shoulder stretching versus shoulder muscle strength training for the prevention of baseball-related arm injuries: a randomized, active-controlled, open-label, non-inferiority study

**DOI:** 10.1038/s41598-022-26682-1

**Published:** 2022-12-21

**Authors:** Hitoshi Shitara, Tsuyoshi Tajika, Takuro Kuboi, Tsuyoshi Ichinose, Tsuyoshi Sasaki, Noritaka Hamano, Masataka Kamiyama, Atsushi Yamamoto, Tsutomu Kobayashi, Kenji Takagishi, Hirotaka Chikuda

**Affiliations:** Department of Orthopaedic Surgery, Gunma University Graduate School of Medicine, 3-39-22, Showa, Maebashi, Gunma 371-8511 Japan

**Keywords:** Rehabilitation, Paediatric research, Risk factors, Randomized controlled trials

## Abstract

Glenohumeral internal rotation deficit (GIRD) and weakness in prone external rotation are risk factors for shoulder and elbow injuries in high school baseball pitchers. While a shoulder-stretching prevention program to improve GIRD decreases the injury rate, the effects of external rotation strength remain unclear. This non-inferiority (NI) study investigates the hypothesis that external rotation strength training is not inferior to sleeper stretching for shoulder and elbow injury prevention in high school baseball pitchers. Participants were randomly allocated to the stretching (n = 62; active control group) and muscle-training (n = 51) groups. Specific exercises were performed each night. Elbow and shoulder injuries were monitored for 150 days. Kaplan–Meier survival curves were generated, and the hazard ratios (HRs) for injury occurrence were calculated using multivariate Cox regression. The log-rank test was used to compare the injury-free time. A one-sided NI test using a fixed NI margin was performed (significance level, *P* = 0.025). The injury rates were 22.6% (n = 14) in the stretching group and 9.8% (n = 5) in the muscle-training group. The muscle-training group had a lower injury rate (*P* < 0.001) and a lower risk of injury than the stretching group (HR = 0.489). Therefore, external rotation muscle strength training is not inferior to stretching for preventing baseball-related arm injuries.

## Introduction

Shoulder and elbow injuries and the resulting pain are major issues for baseball players^1–3^. As reported in a recent systematic review^4^, elbow varus and shoulder external rotation torques at peak external shoulder rotation during pitching^5^, high pitch velocity^6^, glenohumeral internal rotation deficit (GIRD), shoulder external rotation insufficiency^7^, preseason total shoulder rotation deficit, and preseason supraspinatus and prone external rotation strength deficits^8^ are risk factors for shoulder and elbow injuries in professional baseball players. In addition, preseason GIRD^9^, weakness in prone external rotation strength^9^, and supraspinatus weakness^10^ are significant risk factors for injuries in high school baseball players, and pitching > 100 innings in a year^11^, training > 16 h per week^12^, a history of elbow pain^12^, an age of 9–11 years^12^, and playing pitcher or catcher^12^ are risk factors for elbow injuries in youth baseball players.

Although risk factors for shoulder and elbow injuries in baseball players have been relatively well investigated, knowledge regarding prevention strategies is limited. One prospective study^13^ examined the effects of a prevention program including exercises to prevent GIRD and prone external rotation weakness^9^ in high schoolers. Another study reported a significantly lower injury rate among participants who performed sleeper stretching to correct internal rotation deficits than among control participants^13^. However, there was no significant difference in injury rate between pitchers who performed shoulder external rotation strength training and sleeper stretching and the control participants in that study^13^. As the previous study combined external rotation strength training interventions with sleeper stretching, the effectiveness of external rotation strength training remains unknown.

Therefore, this study evaluates the prevention effects of external rotator strength training. Participants were divided into a muscle-training group and an active control group who performed sleeper stretching. Using this non-inferiority (NI) design was necessary as the absence of a proven prevention method in the control group would not be ethical. This study investigates the NI of external rotation strength training to prevent shoulder and elbow injuries in high school baseball pitchers when compared to sleeper stretching.

## Results

**Figure 1:**
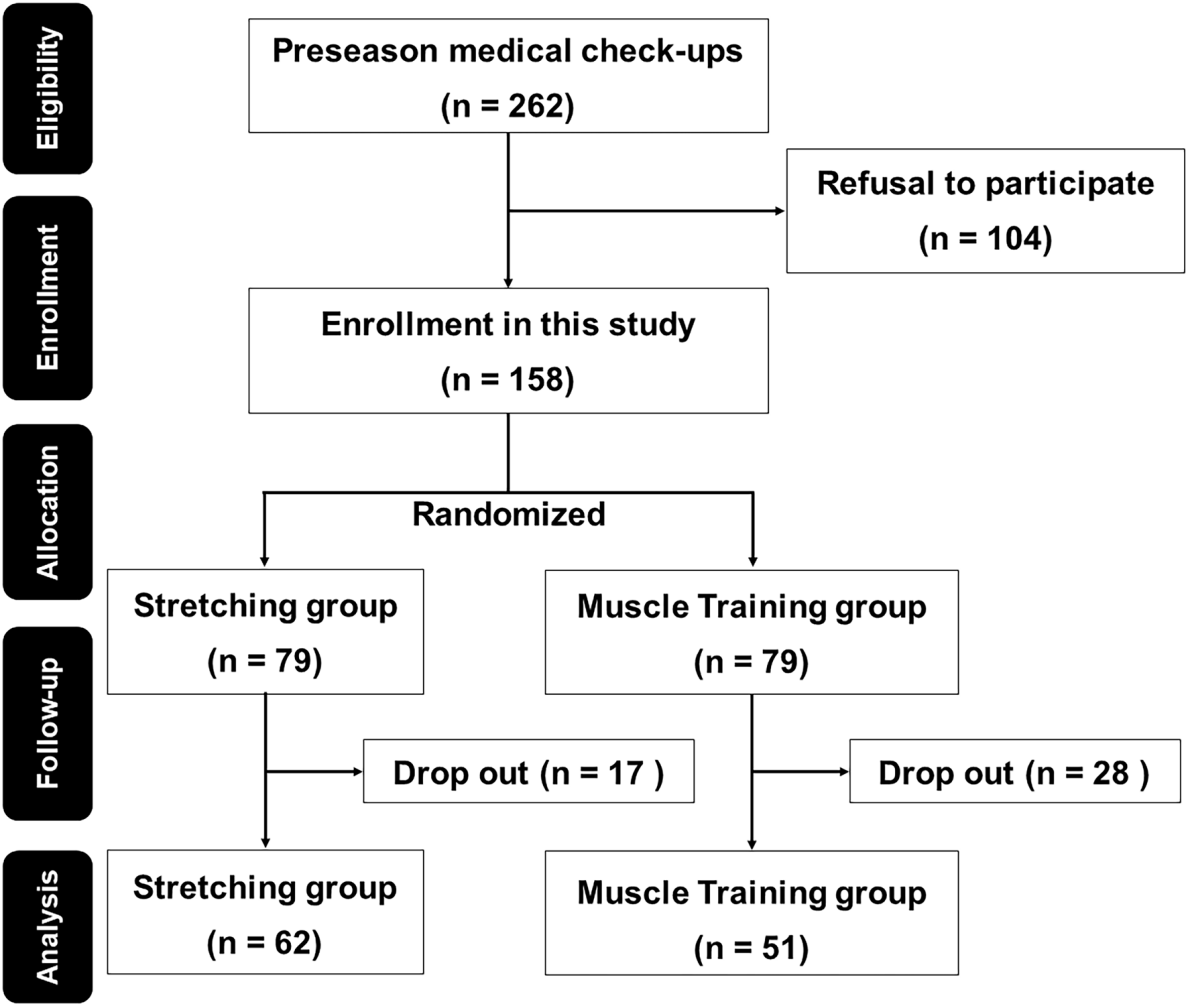
Consolidated Standards of Reporting Trials flow diagram. The Consolidated Standards of Reporting Trials (CONSORT) diagram shows the recruitment, allocation, follow-up, and analysis of the participants.

One hundred fifty-eight pitchers were allocated (1:1) to the stretching and muscle-training groups (Fig. 1). The dropout rate was 21.5% in the stretching group and 35.4% in the muscle-training group (*P* = 0.08). Finally, 62 pitchers in the stretching group and 51 pitchers in muscle-training group were analyzed.

### Baseline characteristics

There were no significant differences in baseball experience during preseason; height; weight; range of motion (ROM) of 90° abduction external and internal rotation (ABER and ABIR, respectively) or elbow extension in the dominant extremity; strength of the supraspinatus in the seated position (SS) and prone external rotation (PER) in the dominant shoulder; ROM differences between the dominant and non-dominant extremities in ABIR, total arc, horizontal adduction (HA), and elbow flexion and extension; strength ratio of SS, PER, or prone internal rotation (PIR); and PIR ratio (PER/PIR). However, the total arc (*P* < 0.001), HA (*P* = 0.001), and elbow flexion on the dominant side (*P* < 0.001) and the difference between the dominant and non-dominant sides in ABER (*P* < 0.001) were significantly lower in the muscle-training group than in the stretching group. The PIR on the dominant side was significantly stronger in the muscle-training group than in the stretching group (*P* = 0.001) (Table 1).

**Table 1:** Baseline characteristics.

Baseline characteristics	Stretching group (n = 62)		Muscle-training group (n = 51)		*P*-value
	Mean	SD	Mean	SD	
Baseball experience (years)	8.3	1.8	8.8	1.6	0.150
Body height (cm)	172.0	5.2	173.8	6.4	0.113
Body weight (kg)	69.2	7.6	69.2	9.1	0.988
**Shoulder ROM**					
ABER on the dominant side (deg)	106.5	15.3	109.1	8.3	0.269
ABIR on the dominant side (deg)	39.6	11.3	37.3	9.0	0.243
Total arc on the dominant side (deg)	162.7	23.3	146.4	12.6	< 0.001*
HA on the dominant side (deg)	18.0	16.1	10.1	8.8	0.001*
Difference in ABER (deg)	34.2	28.9	7.3	11.6	< 0.001*
Difference in ABIR (deg)	− 12.7	13.8	− 10.2	12.6	0.335
Difference in total arc (deg)	− 4.4	15.1	− 2.9	15.7	0.615
Difference in HA (deg)	− 13.3	9.7	− 9.4	13.5	0.080
**Elbow ROM**					
Flexion on the dominant side (deg)	143.4	4.6	138.3	6.7	< 0.001*
Extension on the dominant side (deg)	2.1	5.6	3.1	5.4	0.344
Difference in flexion (deg)	− 1.9	4.0	− 3.0	6.1	0.306
Difference in extension (deg)	− 2.9	5.0	− 3.4	5.2	0.574
**Shoulder strength**					
SS on the dominant side (kg)	9.5	1.5	9.4	1.6	0.728
PER on the dominant side (kg)	13.2	2.1	14.0	2.6	0.107
PIR on the dominant side (kg)	14.5	2.8	16.7	2.6	0.001*
SS ratio	1.0	0.1	1.0	0.1	0.403
PER ratio	1.0	0.2	0.9	0.1	0.058
PIR ratio	1.0	0.2	1.1	0.2	0.226
PER/PIR ratio	0.9	0.2	0.8	0.2	0.060

* *P* < 0.05.

Total arc = ABER + ABIR.

Difference = ROM on the dominant side—ROM on the non-dominant side.

Strength ratio = strength of the dominant/non-dominant side.

PER/PIR ratio = PER/PIR of the dominant side.

ABER and ABIR, ROM in 90° shoulder abduction external and internal rotation, respectively; HA, horizontal adduction; PER, prone external rotation; PIR, prone internal rotation; ROM, range of motion; SD, standard deviation; SS, supraspinatus in the seated position.

### Primary endpoint: NI test for injury rate

**Figure 2:**
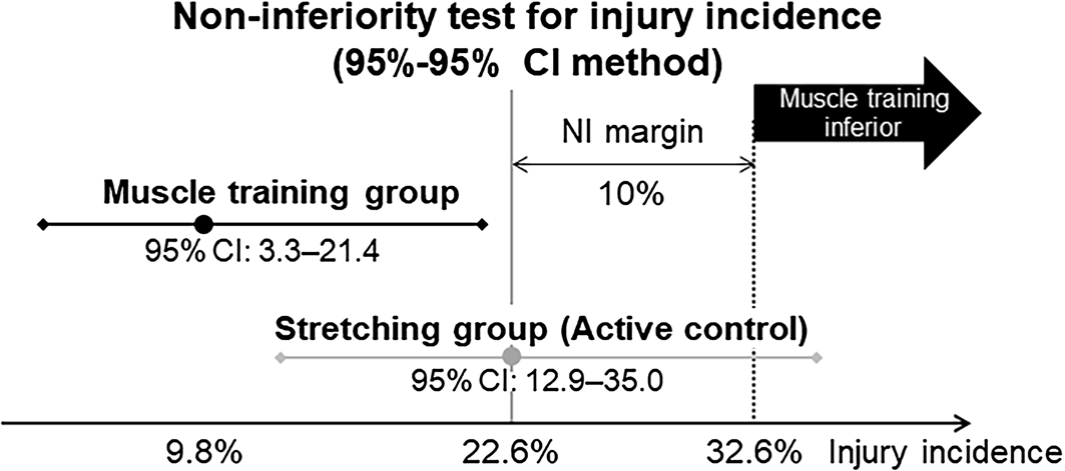
Non-inferiority test for injury rate. Injury rates in the muscle-training and stretching groups were 9.8% (95% confidence interval [CI] 3.3–21.4) and 22.6% (95% CI 12.9–35.0), respectively. The non-inferiority (NI) margin was 10%. The muscle-training group had a significantly lower injury rate than the stretching group with a pre-specified NI margin (*P* < 0.001).

The injury rate was significantly lower in the muscle-training group than in the stretching group (*P* < 0.001, Fig. 2). The post-hoc power analysis value for the NI test was 0.501.

### Secondary endpoint

The injury rate was not significantly different between the groups based on the Fisher’s exact test (*P* = 0.082). The post-hoc power analysis values for Fisher’s exact test and the NI test were 0.329 and 0.501, respectively.

### Time-to-event analysis

**Figure 3:**
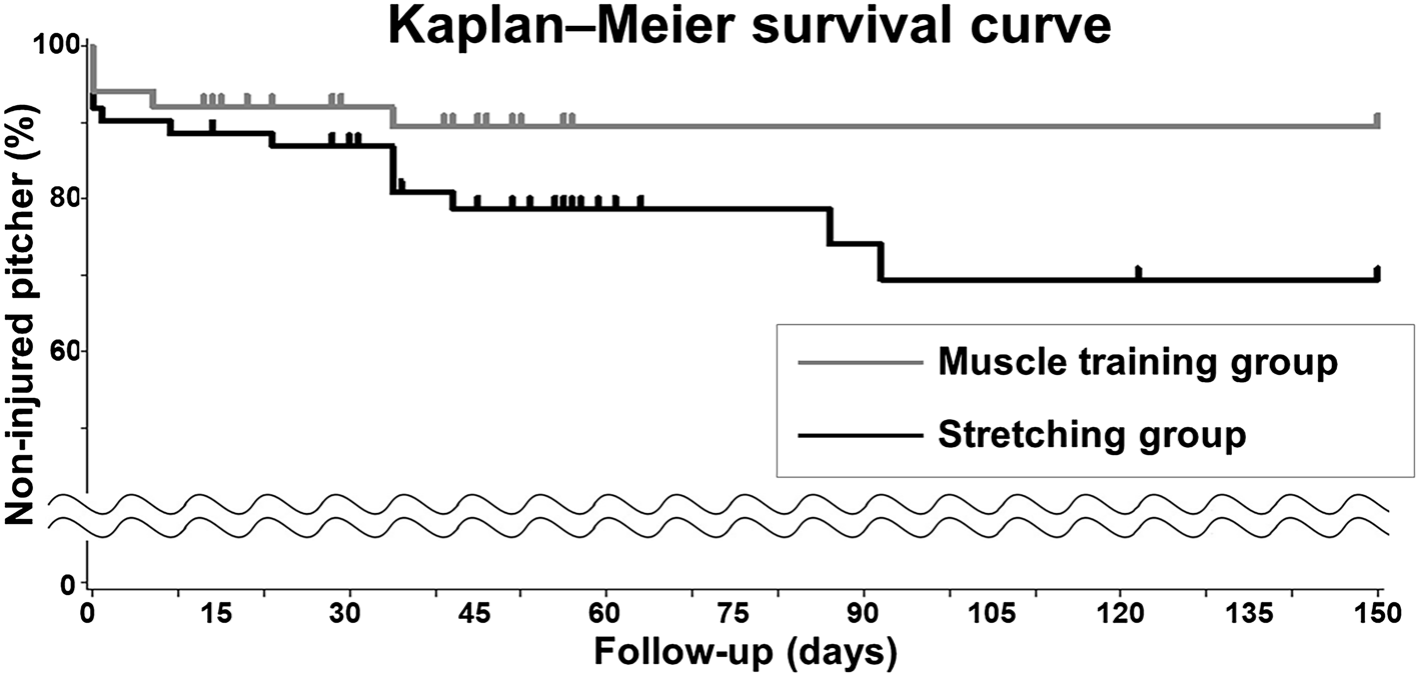
Kaplan–Meier survival curves. The median injury-free times were 52.5 and 49.0 days in the stretching and muscle-training groups, respectively. A log-rank test revealed no significant differences in the injury rates between the stretching and muscle-training groups (*P* = 0.175).

The injury rates in the stretching and muscle-training groups were 22.6% (n = 14) and 9.8% (n = 5), respectively. The median survival times were 52.5 and 49.0 days, respectively (Fig. 3). The muscle-training group had a lower risk of injury than the stretching group (hazard ratio [HR] = 0.489; 95% confidence interval [CI] 0.174–1.375). There was no significant difference in injury rate between the stretching and muscle-training groups (*P* = 0.175). The statistical power was 0.731.

To account for possible bias in important background factors despite randomization, the data were analyzed after correcting for these background factors using a binomial logistic regression and multivariate Cox regression analyses with injury occurrence as the objective variable and the five significantly different factors and group (stretching and muscle-training) as explanatory variables.

No significant explanatory variable was identified (Table 2). The trend in HRs was the same as in the unadjusted models, and no significantly different variables were associated with time to injury occurrence.

**Table 2:** Multivariate analysis and Multivariate Cox regression for testing possible bias.

Multivariate analysis	Odds ratio	95% CI	*P*-value
Group (stretching vs. muscle-training)	1.542	0.382–6.218	0.543
Total arc on the dominant side (deg)	1.013	0.970–1.058	0.558
HA on the dominant side (deg)	1.030	0.984–1.078	0.204
Difference in ABER (deg)	0.984	0.948–1.020	0.372
Elbow flexion on the dominant side (deg)	1.103	0.984–1.238	0.093
PIR on the dominant side (kg)	0.976	0.865–1.100	0.685

Multivariate Cox regression	Hazard ratios	95% CI	*P*-value
Group (stretching vs. muscle-training)	0.729	0.199–2.666	0.633
Total arc on the dominant side (deg)	1.011	0.972–1.050	0.591
HA on the dominant side (deg)	1.020	0.980–1.062	0.326
Difference in ABER (deg)	0.986	0.958–1.015	0.354
Elbow flexion on the dominant side (deg)	1.076	0.972–1.191	0.159
PIR on the dominant side (kg)	0.988	0.888–1.100	0.827

Total arc = ABER + ABIR.

ABER and ABIR, ROM in 90° shoulder abduction external and internal rotation, respectively. HA, horizontal adduction; Difference = ROM on the dominant side—ROM on the non-dominant side; CI, confidence interval; PIR, prone internal rotation.

## Discussion

In this study, external rotation muscle strength training was not inferior to sleeper stretching for the prevention of shoulder and elbow injuries in high school baseball players. This is the first prospective study to provide evidence that external rotation muscle strength training is effective for preventing shoulder and elbow injuries. These results can be used to guide injury prevention programs for baseball-related injuries at any level.

### Shoulder external rotation strength training

Previous prospective studies have reported that a decrease in PER strength is a risk factor for injuries in high school pitchers^9^ and professional baseball players^8^. Therefore, shoulder external rotation strength training was used to maintain and improve the external rotation strength in the participants’ dominant shoulders during the season when the PER strength may decrease due to the number of games. In a previous interventional study regarding injury among high school baseball pitchers^13^, a combined strength training and stretching group exhibited a trend toward a lower incidence of injuries than a control group; however, the difference was not significant (*P* = 0.06). The authors concluded that the muscle strength intervention did not have a significant injury prevention effect, though the study had insufficient statistical power to detect differences between the groups^13^. Insufficient load or inappropriate target muscles may also have contributed to the failure of demonstrating the effectiveness of muscle training for injury prevention as the load used during external rotation training was 500 g and the inner muscles, including the infraspinatus and teres minor, were targeted. PER weakness is typically measured using maximum power generated by the inner and outer muscles.

Dark et al.^14^ compared the maximum voluntary isometric contractions (MVICs) in the infraspinatus, supraspinatus, and posterior deltoid muscles between healthy volunteers performing low-, medium-, and high-intensity external rotation exercises of the shoulder using electromyography. As the load intensity increased, the infraspinatus muscle activity increased from 40 of the MVIC to 70% of the MVIC. Similar increase patterns were observed in the supraspinatus and posterior deltoid muscles (supraspinatus muscle: 15% of the MVIC to 51% of the MVIC; posterior deltoid muscle: 6% of the MVIC to 31% of the MVIC), and the MVIC in the infraspinatus was the highest at all load intensities. Therefore, when a low load is used, the main target muscle is the supraspinatus, and when a high load is used, the infraspinatus, supraspinatus, and posterior deltoids will be trained. In this study, isometric training using the maximum power strength was used. Although the mechanisms by which muscle strength training reduces injuries remain unclear, training may either maintain or improve shoulder external rotation strength during the baseball season and reciprocal inhibition by the contraction of muscles involved in external rotation of the shoulder may induce relaxation of the muscles involved in internal rotation, improving or maintaining ABIR.

### NI test for injury incidence

A non-interventional group was not included in this study due to ethical concerns, as a previous interventional study^13^ reported a very high injury rate in the non-interventional group (57%). This study used an NI test with an active control condition and a stretching exercise that has been reported as an effective intervention for reducing injury^13^.

External rotation muscle strength training was not inferior to sleeper stretching for the prevention of shoulder and elbow injuries in this study. The optimal NI margin was difficult to determine as evidence from previous prospective studies of injury prevention via self-exercise is limited. In this study, the injury rate was significantly lower in the muscle-training group than in the stretching group using a pre-specified NI margin of less than 10% (i.e., any NI margin).

Although superiority testing was not the purpose of this study, the superiority of external rotation muscle strength training to sleeper stretching was not demonstrated due to the small sample size (post-hoc power = 0.329).

As the power analysis, performed using the parameters obtained in this study, required 145 participants in each group, further investigation with larger samples is required in the future.

### Baseline characteristics

Previous prospective studies identified a significant decrease in ABIR on the dominant side^9^, large deficits in ABIR^15^, a significant decrease in the dominant/non-dominant ratio of PER strength^9^, and SS weakness during preseason^10^ as significant risk factors for elbow and shoulder injuries in high school baseball pitchers. Baseline data in this study revealed no significant differences in these risk factors.

Currently, there is no consensus regarding whether total arc and HA are risk factors for injury in high school baseball pitchers^9,15,16^. In this study, total arc and HA on the dominant side were significantly smaller in the muscle-training group than in the stretching group. The laterality between the dominant and non-dominant sides was not significantly different, suggesting that its effect on injury incidence in this study is minimal.

### Limitations

This study is not without limitations. First, it is unclear how muscle strength training prevents injuries as no physical examinations or imaging were conducted in this study. Second, data regarding the severity of injuries were not included, and no follow-up data were recorded. As this study included volunteers, a follow-up period could not be enforced. Third, the dropout rates were relatively high in both groups due to the strict dropout criteria, and the timing of dropout was not analyzed. Some participants may have found the protocols tedious. Although the dropout rate might have affected the results, such influence may not have been sufficient to alter the outcome as there was no significant difference in the dropout rate between the groups. Finally, the groups were not balanced for measured confounders despite simple randomization, which may have affected the results. Although minimization or stratified block randomization may have been necessary, additional analyses revealed that baseline differences did not affect the results. Additionally, the randomization likely helped reduce bias of unmeasured confounders, such as the amount of pitches and practices. To reduce the bias related to the timing of baseball games, the participants played in the same baseball league in the same district and had similar tournament schedules. These limitations should be addressed in future studies.

## Conclusions

External rotation muscle strength training for the prevention of baseball-related arm injuries is not inferior to sleeper stretching in high school baseball pitchers. This is the first study to provide evidence that external rotation muscle strength training can prevent shoulder and elbow injuries. These results should be further validated in future studies to guide injury prevention programs for baseball-related injuries.

## Methods

### Study design

This is a randomized, active-controlled, open-label, NI study with a level of evidence of 1.

### Participants

Preseason medical check-ups were conducted in February 2015 for 262 male high school baseball pitchers aged 15–17 years. During these check-ups, the pitchers were invited to participate in this study. Written informed consent was obtained from the parents of all participants. As 104 pitchers were not interested in participating, 158 were enrolled in the 150-day prospective study. Using previously reported inclusion criteria^9,13^, all participants were active pitchers during preseason workouts and had no restrictions on their pitching activities. Pitchers with prior injuries (such as fractures) to the throwing arm, those who could not throw or had restricted pitching activity due to a shoulder or elbow problem, and those who performed daily muscle-specific rotator cuff training exercises or posterior capsule/sleeper stretches outside of team exercises were excluded from the study^9,13^.

The Institutional review board of Gunma University Hospital (identification number 1003) approved this study. All methods were performed in accordance with the relevant guidelines and regulations.

#### Randomization

A computer-generated random number table was created using the RAND function of Excel (Microsoft, Washington, U.S.) and participants were collated using anonymous identification numbers. The participants were randomly assigned to the muscle-training or the stretching group at a ratio of 1:1. Randomization was performed via the simple randomization method using a random number table. The random allocation sequence was concealed from a person assigning participants to the intervention groups until patient consent was obtained.

### Medical check-ups

As previously reported^9,13^, preseason medical check-ups were performed to evaluate the preseason condition of the participants’ shoulders and elbows and to prevent shoulder and elbow injuries. To avoid confirmation bias, the examiners were not aware of the participants’ hand dominance. The participants’ body weight, height, baseball experience, shoulder and elbow ROM, and shoulder muscle strength were evaluated.

### Shoulder and elbow ROM

Using a digital protractor (iGaging, Los Angeles, CA, USA), a certified orthopedic surgeon measured the passive elbow flexion and extension ROM^9,13^, passive HA, ABER, and ABIR ROM on the dominant and non-dominant sides. All measurements were performed in the supine position. When passive HA was measured, the examiner stabilized the axillary border of the scapula and another certified orthopedic surgeon placed a digital protractor on the humerus. When passive ABER and ABIR were measured, the examiner stabilized the scapula by applying posterior force to the coracoid process and another certified orthopedic surgeon placed a digital protractor on the forearm. The total arc was calculated for the dominant and non-dominant shoulders by adding 90° ABER and ABIR for each shoulder. Furthermore, the difference in each measurement was calculated as ROM on the dominant side minus ROM on the non-dominant side.

### Shoulder strength

Using a PowerTrack II Commander hand-held dynamometer (J-Tech Medical, Salt Lake City, UT, USA), a certified orthopedic surgeon measured the strength of the SS, PIR, and PER in both shoulders^9,13^. When SS strength was measured, the participants were asked to sit on an examination table with their back against the wall and to abduct the humerus to 90° in the coronal plane and then adduct horizontally to 45° with the forearm in a neutral position. The dynamometer was placed 5 cm proximal to the proximal wrist extension crease as the pitcher raised his arm perpendicular to the floor with maximum effort. The PIR and PER strengths were measured in the supine position. Prior to the measurement, the participants were instructed to abduct the humerus and flex the elbow to 90°. Then, the humerus was stabilized by the examiner, and the arm was set in a neutral position. The participants rotated their arm externally or internally with maximum power against the dynamometer. When PIR strength was measured, the dynamometer was placed on the volar side of the forearm, 5 cm proximal to the proximal wrist flexion crease. When PER strength was measured, the dynamometer was placed on the dorsal side of the forearm, 5 cm proximal to the proximal wrist extension crease.

Each measurement was repeated three times and recorded. The median data values were analyzed. The ratios of SS, PER, and PIR strength in the dominant to non-dominant arms, and the ratio of dominant arm PER to dominant arm PIR strength, were calculated for each participant.

### Prevention program

Ten physical therapists educated the participants regarding the prevention program exercises. To avoid differences in the instructions, each therapist was trained by the same orthopedic surgeon (H.S.) who regularly monitored the delivery of the instructions to the study participants. A brochure illustrating the stretching or strength training exercises and written summaries of the instructions was provided.

**Figure 4:**
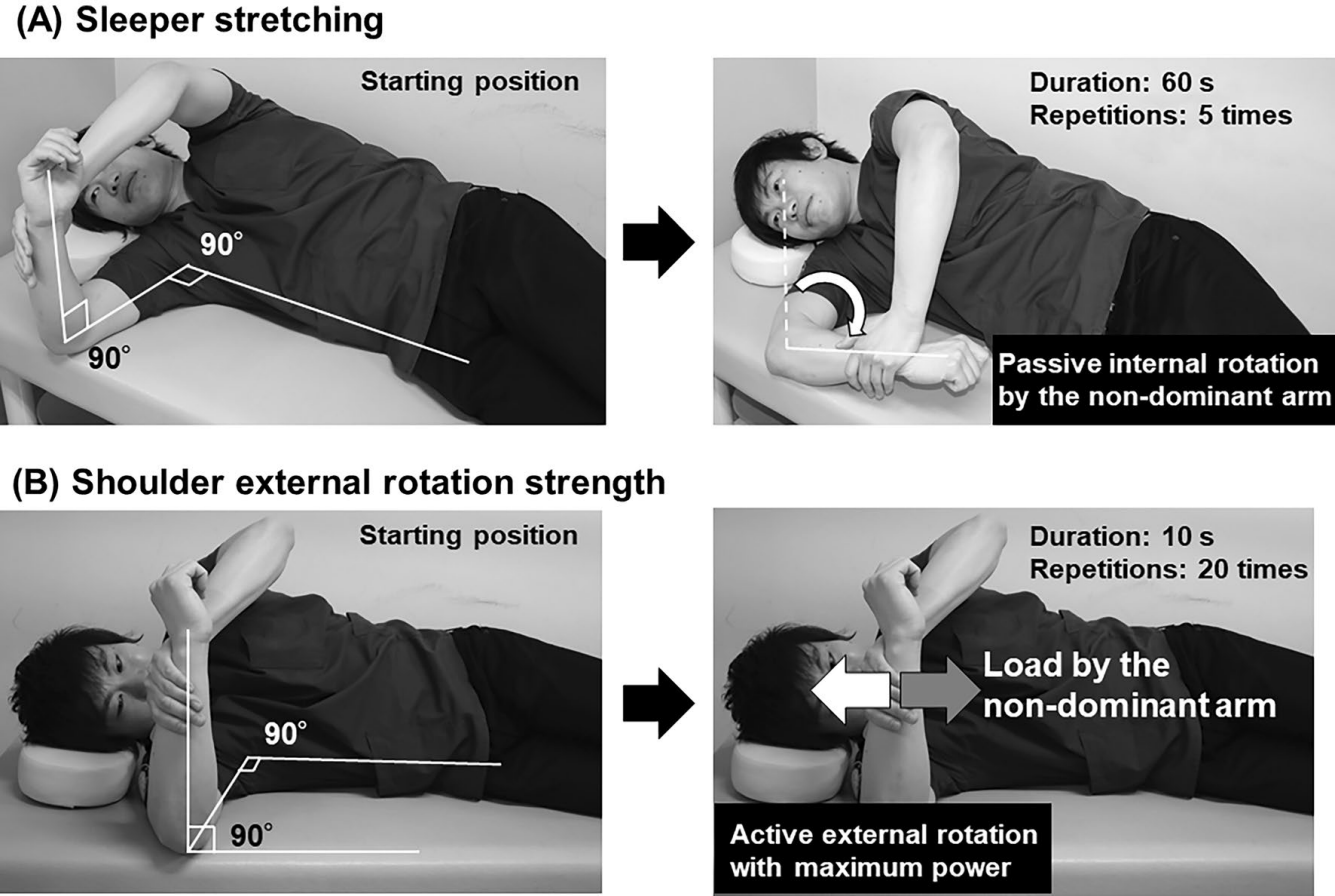
Prevention programs. (**a**) Sleeper stretching exercises: (1) The starting position is a dominant lateral position with the dominant scapula in contact with the bed, the dominant shoulder flexed to 90°, the dominant elbow flexed to 90°, and the dominant forearm in a neutral position. (2) The dominant forearm is grasped proximal to the dominant wrist with the non-dominant hand. (3) The dominant shoulder is passively and internally rotated by the non-dominant hand to the end of its maximum range of motion and that position is held for 1  min. (4) After a 30-s break, the stretching is repeated for a total of five times each night. (**b**) Shoulder external rotation strengthening exercises: (1) The starting position is the same as that of the sleeper stretch. (2) The dominant shoulder is maximally externally rotated for 10 s against an internal rotation force by the contralateral arm (isometric exercise). (3) After a 10-20-s break, the training is repeated for a total of 20 times each night.

The physical therapists provided 30 min of one-on-one instruction regarding self-stretching or strength training exercises with each participant immediately after allocation. Each participant received the brochure regarding the instructions and an overview of compensatory movements to avoid (Fig. 4). The participants were instructed to perform the assigned exercise program once daily after baseball practice.

### Sleeper stretching as an “active control” condition

To improve the dominant shoulder internal rotation deficit, participants were asked to perform a daily sleeper stretch^13,17,18^ with the dominant shoulder during the baseball season (five repetitions of 60 s each). The sleeper stretching exercise is illustrated in Fig. 4a. As this stretching program significantly decreased the incidence of shoulder and elbow injuries in a previous study^13^, it was used as an active control in the current study.

### Shoulder external rotation strength training

A modified strength training protocol focusing on the inner shoulder muscles, including the infraspinatus and teres minor^9^, and on the outer shoulder muscles, including the deltoid and the trapezius, was used as both the inner and outer muscles contribute to external rotation strength.

The shoulder external rotation strengthening exercise is illustrated in Fig. 4b. The starting position is the same as that for the sleeper stretch. The dominant shoulder is maximally externally rotated for 10 s against an internal rotation force by the contralateral arm (isometric exercise). After a short break of 10–20 s, the training is repeated 20 times every night.

### Injury tracking

In this study, a shoulder or elbow injury was defined as any condition resulting in the pitcher being considered disabled for at least 8 days, excluding injuries due to external forces, such as being hit by a ball, colliding with another player, or falling^9,13,19–27^. Participants completed a questionnaire regarding the presence of shoulder and/or elbow pain, limitations to pitching caused by shoulder or elbow pain, and the presence of other injuries each day to record the timing of injuries and avoid recall bias. The participants’ responses were submitted to the researchers monthly. Furthermore, to confirm that the participants completed the daily questionnaires, they were contacted via telephone once or twice per month. Dropout was defined as when the participants did not complete the questionnaire once or when the compliance rate of the assigned exercise program was lower than 66.6% as this study included a time-to-event analysis.

### Statistical analyses

All statistical analyses were conducted using SAS version 9.4 (SAS Institute Inc., Cary, NC, USA). All tests, with the exception of the NI test, were two-sided with a significance level of *P* = 0.05.

#### Sample size

An a priori statistical power analysis indicated that 79 participants were required in each group to provide a statistical power of 80% at an α level of 0.05 based on a one-sided test with an injury incidence of 57.1% in the muscle-training group and 25.0% in the stretching group^13^, a dropout rate of 20%, and an NI margin of 10%.

#### Baseline characteristics

The inter-group differences in dropout rate and injury incidence were evaluated using Fisher’s exact test. Baseline characteristics are reported as mean ± standard deviation. The Mann–Whitney U test was used to detect differences in baseline characteristics between the groups.

#### Primary endpoint analyses

To demonstrate the NI of muscle-training to the stretching exercises, the NI test was performed using the 95%–95% CI fixed-margin method. A restrictive NI margin of 10%, traditionally used in clinical NI trials^28^, was used as prior evidence for the prevention of baseball-related arm injuries was limited to determine an appropriate NI margin. Therefore, the upper limit of the 95% CI for injury incidence difference was set at 10% or lower (the pre-specified NI margin for this endpoint). Statistical significance for the NI test was set at *P* = 0.025.

#### Secondary endpoint analyses

The secondary endpoint was the superiority of muscle-training and stretching for injury prevention. Fisher’s exact test was performed to evaluate whether muscle-training was superior to stretching for injury prevention. Consequently, the Kaplan–Meier method was used to obtain time-to-event curves, and HRs were calculated for the injury rates using Cox proportional hazard models. To compare survival distributions between the groups, a log-rank test was performed.

After data collection, a post-hoc power analysis was conducted to determine the statistical power of the study.

### Ethics approval and consent to participate

Informed consent was obtained from the participants’ parents.

## Data Availability

The data supporting the findings of this study are available from the corresponding author (HS) by request. The data are not publicly available as they contain information that could compromise the privacy of the participants.
